# Maternal and fetal outcomes in pregnancies complicated by overweight and obesity

**DOI:** 10.1186/s12978-016-0206-0

**Published:** 2016-08-27

**Authors:** Joice Monaliza Vernini, Jusciele Brogin Moreli, Claudia Garcia Magalhães, Roberto Antônio Araújo Costa, Marilza Vieira Cunha Rudge, Iracema Mattos Paranhos Calderon

**Affiliations:** 1 Graduate Program in Gynecology, Obstetrics and Mastology, Botucatu Medical School, São Paulo State University - Unesp, São Paulo, Brazil; 2 Department of Obstetrics and Gynecology, Botucatu Medical School, São Paulo State University – Unesp, São Paulo, Brazil

**Keywords:** Obesity, Overweight, Pregnancy, Maternal outcomes, Perinatal outcomes

## Abstract

**Background:**

Overweight and obesity are associated with pregnancy complications and adverse perinatal outcomes, posing short and long**-**term risks for maternal and child health. This study evaluated maternal, delivery and neonatal outcomes in pregnancies complicated by overweight and obesity.

**Methods:**

This prospective cross-sectional study included 258 pregnant women. According to prepregnancy body mass index (BMI), participants were classified as normal weight, overweight, or obese. Data were analyzed using the chi-square test and analysis of variance followed by the Tukey test. Logistic regression was performed to calculate odds ratios and 95 % confidence intervals (*p* < 0.05).

**Results:**

Most women ≥ 35 years old were overweight (22.7 %) and obese (27.6 %). Prepregnancy diabetes was significantly associated with obesity (15.7 %, *p* < 0.000). Obese women showed the lowest weight gain (9.6 ± 7.5Kg). Overweight and obese women practiced physical exercise more frequently (*p* = 0.010) than normal weight women. A greater proportion of obese mothers (13.4 %) had large for gestational age babies (*p* = 0.021), with higher thoracic circumference (33.6 ± 2.0 cm) and abdominal circumference (31.6 ± 2.3 cm). Obesity increased the risk of developing hypertension (OR = 7.0; 3.1-15.9), hyperglycemic disturbances (OR = 5.5; 2.9-10.6) and HbA1c ≥ 6.5 % (OR = 3.7; 1.2-11.1). The infants born to obese mothers had longer hospital stay (3.9 ± 3.9 days) (*p* = 0.005).

**Conclusion:**

Our results confirm that obesity in pregnancy can lead to adverse outcomes, and underscore the importance of identifying and treating inadequate weight status during pregnancy.

## Background

Overweight and obesity are defined as abnormal or excess accumulation of adipose tissue in the body. These conditions are caused by a combination of genetic, metabolic, behavioral, environmental, cultural, and socioeconomic factors. Overweight and obesity are public health problems that contribute to preventable deaths each year, and the number of overweight and obese women at reproductive age is increasing in many countries [1, 2].

Since pregnancy can serve as a triggering or aggravating factor for obesity [3, 4], diagnosing and monitoring the weight status of pregnant women should be a routine prenatal care procedure [5, 6]. A number of factors, such as water retention, uterine growth, formation of fetal tissues and placenta, and increasing amniotic fluid volume, can limit the evaluation of maternal body mass index (BMI) during pregnancy [7, 8]. Therefore, since 2004, the Brazilian Ministry of Health has issued guidelines for categorizing ideal gestational weight gain based on prepregnancy BMI. These guidelines, recommend assessing a woman’s nutritional status during pregnancy using a specific tool [6] which was developed based on Atalah’s curve [9] and IOM recommendations [8].

Overweight pregnant women have higher risks of hypertension, gestational diabetes mellitus (GDM), Cesarean section, shoulder dystocia and early neonatal death [10–14]. Obesity in pregnancy compromises fetal metabolic programming, increasing the risk of obesity, diabetes and cardiovascular disorders in the offspring [15, 16].

Thus, the present study aimed at evaluating maternal, delivery and neonatal outcomes in pregnancies complicated by overweight and obesity.

## Methods

### Study design and subjects

This is a prospective cross-sectional study including 258 pregnant women and their infants. Based on prepregnancy weight as self-reported during the first prenatal visit, pregnant women were classified according to BMI as normal weight (18.5–24.9 Kg/m^2^ BMI; *n* = 65); overweight (25.0 - 29.9 Kg/m^2^ BMI; *n* = 66); or obese (BMI ≥ 30.0 Kg/m^2^; *n* = 127) [17].

Ethics approval was granted by the Human Research Ethics Committee of Botucatu Medical School-Unesp, and written informed consent was obtained from all study participants.

### Inclusion and exclusion criteria

Inclusion criteria were prenatal care commencing up to 20 weeks of gestationand singleton pregnancy. Underweight pregnant women were excluded.

### Data collection

During the first prenatal visit (before 20th gestational week), the following maternal characteristics were evaluated: age (full years); number of gestations including current pregnancy; previous Cesarean section; smoking habits, history of hypertension and diabetes mellitus,and family history of diabetes, obesity, hypertension, cardiovascular disease and hypercholerestolemia. At the end of gestation (37th to 38th gestational week), maternal pregnancy outcomes were evaluated based on final pregnancy weight; gestational weight gain; gestational BMI; practice of light to moderate-intensity exercises during pregnancy (yes or no); and intercurrent diseases (such as urinary infection, genital infection, gestational hypertension, preelampsia, hyperglycemic disorders (mild gestational hyperglycemia – MGH, and gestational diabetes mellitus – GDM) [18, 19], and glycated hemoglobin (HbA1c) levels in the third trimester of pregnancy.

Data collected at birth included mode of delivery (vaginal or C-section); gestational age; New Ballard scores; birth weight; birth weight/gestational age category; ponderal index [(birth weight/lenght^3^) × 100]; length; circumference of the head, thorax, and abdomenl; 1, 5 and 10 min Apgar scores; placenta weight; and placental index (PI = placenta weight/birth weight). In the cord blood, the levels of glucose, hematocrit, hemoglobin, bilirubin, and white and red blood cells were evaluated.During the neonatal period, information on hypoglycemia episodes; need for phototherapy; malformations and infant hospital stay length was obtained.All data were recorded on forms specifically developed for the study.

### Follow-up

All women received follow up care at the Diabetes and Pregnancy Referral Center of Botucatu Medical School-Unesp. Reasons for referral were previous diabetes mellitus (type 1-DM or type 2-DM) or risk of developing hyperglycemic disorders [20], i.e. gestational diabetes mellitus (GDM) and mild gestational hyperglycemia (MGH) [17, 19].

According to our center’s routine protocol [18, 19], diabetic pregnant women (type 1-DM or type 2-DM) were immediately managed with glycemic control, individualized nutritional intervention, and a light to moderate-intensity exercise program (most frequently walking for 30 minutes five times a week). Insulin therapy was introduced when necessary [21].

Pregnant women who were nondiabetic, but were overweight or obese, were advised as to the importance of lifestyle changes to prevent GDM and MGH, and were promptly assigned to individualized nutritional guidance and home walking for 30 minutes five times a week for weight control during pregnancy. Regardless of these preventive measures, all nondiabetic pregnant women underwent glucose tolerance (75 g-OGTT) and gycemic profile (GP) testing between 24 and 28 weeks of pregnancy for confirmation or ruling out of GDM and MGH [18–20]. Pregnant women with confirmed GDM or MGH were treated according to the same protocol to achieve glycemic control. Insulin therapy was introduced when necessary. Glycemic control and management of diabetes were evaluated by 24-h GP (fasting, pre- and post- prandial glycemic levels) performed at 2-week intervals until week 32, and weekly until delivery [18–20].

### Statistical analyses

Data were systematically transferred from medical records to Microsoft Excel 2010. Statistical analyses were performed using SAS 9.2 for Windows. Data frequencies (in percentage) were compared using the chi-square test; means were evaluated by analysis of variance (ANOVA), followed by the Tukey-Kramer test for multiple comparisons. Data on maternal, neonatal and delivery outcomes were assessed using logistic regression to calculate odds ratios with confidence intervals (CI 95 %). Significance level was set at *p* < 0.05.

## Results

Overweight and obesity predominated among pregnant women ≥ 35 years, whereas normal weight was most frequently observed among pregnant adolescents (≤19 years old) (*p* < 0.001). Prepregnancy diabetes was more frequent in obese (15.7 %) and overweight (13.6 %) women than in women with normal weight (6.2 %). The prevalence of of family history of obesity was greater in obese and overweight women, although not statistically significant (*p* = 0.051) (Table 1).

**Table 1:** Characteristics of the participating pregnant women

	Normal weight *N* = 65	Overweight *N* = 66	Obese *N* = 127	*p*-value*
	N (%)	N (%)	N (%)	
Age (years)*				**<0** **.00** **1**
≤19	12 (18.5)	**2 (3.0)** ^**a**^	**1 (0.8)** ^**a**^	
20 – 35	46 (70.8)	49 (74.2)	91(71.6)	
>35	7 (10.8)	**15 (22.7)** ^**a**^	**35 (27.6)** ^**a**^	
Pregnancies (including current pregnancy)				0.187
1	18 (27.7)	11 (16.7)	19 (15.0)	
2	19 (29.2)	18 (27.3)	33 (26.0)	
≥3	28 (43.1)	37 (56.1)	75 (59.6)	
Previous C-section				0.366
Zero	44 (67.7)	35 (53.0)	68 (53.5)	
1	14 (21.5)	20 (30.3)	36 (28.4)	
≥2	7(10.8)	11 (16.7)	23 (18.1)	
Smoking habits	9 (13.8)	17 (25.8)	20 (15.8)	0.141
Cronic hypertension	4 (50.0)	17 (81.0)	47 (74.6)	0.231
Prepregnancy diabetes	4 (6.2)	**9 (13.6)** ^**a**^	**20 (15.7)** ^**a**^	**<0.001**
Family history				
Diabetes	54 (83.1)	48 (72.7)	93 (73.2)	0.266
Obesity	17 (26.2)	24 (36.4)	**56 (44.1)** ^**a,b**^	**0.051**
Hypertension	44 (67.7)	49 (74.2)	101 (79.5)	0.195
Cardiovascular disease	14 (21.5)	19 (28.8)	43 (33.9)	0.206
Hypercholesterolemia	15 (23.1)	20 (30.3)	41 (32.3)	0.410

* Chi-square test

^a^ statistically different from the control group (*p* < 0.05)

^b^ statistically different from the overweight group (*p* < 0.05)

Compared to women with normal weight, overweight and obese women showed higher mean baseline weight and final weight, prepregnancy BMI and gestational BMI (at 37–38 weeks of pregnancy) (*p* < 0.001). Nonetheless, weight gain (WG) was lowest in the obese group (Table 2).

**Table 2:** Maternal outcomes

	Normal weight *N* = 65	Overweight *N* = 66	Obese *N* = 127	*p-value
	Mean ± sd	Mean ± sd	Mean ± sd	
Initial weight (Kg)	57.1 ± 5.7	**69.9 ± 6.7** ^**a**^	**91.2 ± 13.7** ^**a,b**^	**<0.001**
Final weight (Kg)	71.5 ± 7.1	**82.9 ± 7.6** ^a^	**100.7 ± 15.4** ^**a,b**^	**<0.001**
Weight gain (Kg)	14.4 ± 4.6	12.8 ± 5.7	**9.6 ± 7.5** ^**a,b**^	**<0.001**
Prepregnancy BMI (Kg/m^2^)	22.4 ± 1.7	**27.5 ± 1.3** ^**a**^	**36.2 ± 4.9** ^**a,b**^	**<0.001**
Gestational BMI (Kg/m^2^)	28.1 ± 2.4	**32.6 ± 2.7** ^**a**^	**39.9 ± 5.5** ^**a,b**^	**<0.001**
	N (%)	N (%)	N (%)	†*p*-value
Weight gain (Kg)				**<0.001**
<8	5 (7.8)	12 (18.2)	**58 (45.7)** ^**a,b**^	
8 – 16	36 (55.4)	36 (54.6)	**42 (33.1)** ^**a,b**^	
>16	24 (36.9)	18 (27.3)	27 (21.3)	
Gestational BMI (Kg/m^2^)				**<0.001**
Underweight	7 (10.8)	**0 (0.0)** ^a^	**0 (0.0)** ^**a**^	
Adequate	33 (50.8)	**5 (7.6)** ^a^	**0 (0.0)** ^**a,b**^	
Overweight	23 (35.4)	**35 (53.0)** ^a^	**8 (6.3)** ^**a,b**^	
Obese	2 (3.1)	**26 (39.4)** ^a^	**119 (93.7)** ^**a,b**^	
Physical activity (yes)	17 (26.2)	**34 (51.5)** ^a^	**55 (43.3)** ^a^	0.010
Intercurrent diseases	30 (46.2)	26 (39.4)	62 (48.8)	0.458
Urinary infection	8 (12.5)	8 (11.9)	30 (23.6)	
Genital infection	12 (18.8)	12 (17.9)	15 (11.8)	
GH or PE	4 (50.0)	4 (19.0)	16 (25.4)	
GDM	8 (12.3)	**22 (33.3)** ^**a**^	**50 (39.4)** ^**a**^	**<0.001**
MGH	12 (18.4)	**15 (22.7)** ^**a**^	**27 (21.2)** ^**a**^	**0.005**
HbA1c (%) [3rd trimester]				0.075
<6.5	61 (93.8)	53 (80.3)	102 (80.3)	
6.5 – 8.0	3 (4.6)	13 (19.7)	23 (18.1)	
≥8.0	1 (1.6)	0 (0.00)	2 (1.57)	

* ANOVA; Tukey test

† Chi-square test

^a^ statistically different from the control group (*p* < 0.05)

^b^ statistically different from the overweight group (*p* < 0.05)

WG < 8 Kg was more frequent among the obese, while excess WG (WG > 16 kg) was more frequently seen in women with normal weight (*p* < 0.001). By the end of pregnancy, most normal weight women were either overweight (35.4 %) or obese (3.1 %) (*p* < 0.001). Most overweight and obese women engaged in physical activity during pregnancy (*p* = 0.010), and developed either GDM (*p* < 0.001) or MGH (*p* = 0.005). Irrespective of group, over 80.0 % of the pregnant women with hyperglycemia exhibited normal HbA1c levels (<6.5 %) in the third trimester of pregnancy, with no differences among groups (*p* = 0.075) (Table 2).

Obese women had the highest rate of LGA (13.4 %) and the lowest rate of SGA infants (4.7 %) (*p* = 0.021); with 40.9 % of these infants showing disproportionate growth (IP ≥ 2.98) (*p* = 0.037). Mean thoracic circumference (*p* = 0.006), abdominal circumference (*p* = 0.032), placental weight (*p* < 0.001) and placental index (*p* = 0.037) were higher in the obese group (Table 3). The infants born to obese mothers remained in hospital for a longer period (*p* = 0.005), but maternal weight status did not affect neonatal outcomes (Table 4).

**Table 3:** Delivery and neonatal outcomes

	Normal weight	Overweight	Obese	*p-value
	*N* = 65	*N* = 66	*N* = 127	
	N (%)	N (%)	N (%)	
Mode of delivery				0.304
Vaginal	25 (39.5)	21 (31.8)	35 (27.6)	
Cesarean	40 (61.5)	45 (68.2)	92 (72.4)	
Gestational age (weeks)				0.494
<37	3 (4.6)	5 (7.6)	12 (9.4)	
≥37	62 (95.4)	61 (92.4)	115 (90.6)	
New Ballard (weeks)±				0.115
<37	3 (5.8)	6 (10.9)	19 (17.1)	
≥37	49 (94.2)	49 (89.1)	92 (82.9)	
Birth weight (g)				0.304
<2500	4 (6.2)	2 (3,0)	2 (1.6)	
2500 − 4000	57 (87.7)	61 (92.4)	113 (89.0)	
≥4000	4 (6.2)	3 (4.6)	12 (9.4)	
Weight class				**0.021**
SGA	8 (12.3)	10 (15,2)	**6 (4.7)** ^**a,b**^	
AGA	54 (83.1)	53 (80.3)	104 (81.9)	
LGA	3 (4.6)	3 (4.6)	17 (13.4)	
Ponderal index (PI)				**0.037**
<2.98 (proportional)	50 (76.9)	46 (69,7)	75 (59.1)	
≥2.98 (disproportionate)	15 (23.1)	20 (30.3)	**52 (40.9)** ^**a,b**^	
	Mean ± sd	Mean ± sd	Mean ± sd	#*p*-value
Weight (g)	3210.2 ± 469.2	3208.9 ± 430.4	3365.9 ± 483.4	0.199
Length (cm)	48.6 ± 2.2	48.4 ± 2,0	48.6 ± 2.1	0.759
Head Circumf (cm)	34.4 ± 1.5	34.4 ± 1,3	34.8 ± 1.5	0.094
Thoracic Circumf (cm)	32.9 ± 1.9	32.8 ± 1,7	**33.6 ± 2.0** ^**a,b**^	**0.006**
Abdom Circumf (cm)	31.1 ± 2.3	30.7 ± 1,8	**31.6 ± 2.3** ^**a,b**^	**0.032**
1-min Apgar	8.3 ± 1.4	8.2 ± 1,8	7.8 ± 1.5	0.076
5-min Apgar	9.3 ± 0.8	9.3 ± 0.9	9.1 ± 0.8	0.143
10-min Apgar	9.7 ± 0.5	9.6 ± 0.6	9.5 ± 0.6	0.071
Ponderal index	2.8 ± 0.3	2.8 ± 0.3	**2.9 ± 0.3** ^**a,b**^	**0.005**
Placental weight (g)	560.1 ± 138,9	574,6 ± 119,8	**635,6 ± 156,6** ^**a,b**^	**0.001**
Placental index	0.2 ± 0.0	0.2 ± 0.0	**0.2 ± 0.0** ^**a,b**^	**0.037**

± *N* = 217 [41 newborns with incomplete data]

* Chi-square test

# ANOVA; Tukey test

^a^ statistically different from the control group *p* < 0.05)

^b^ statistically different from the overweight group (*p* < 0.05)

**Table 4:** Neonatal outcomes^±^

	Normal weight *N* = 65	Overweight *N* = 66	Obese *N* = 127	*p-value
	N (%)	N (%)	N (%)	
Hypoglycemia	0	0	1 (1.0)	0.620
Malformation	2 (4.4)	2 (4.0)	6 q	0.833
Phototherapy	14 (23.0)	10 (16.4)	37 (37.0)	0.091
	Mean ± SD	Mean ± SD	Mean ± SD	#*p*-value
Hematocrit (%)	48.3 ± 5.3	51.0 ± 9.3	49.6 ± 5.4	0.163
Hemoglobin (g/dL)	16.2 ± 1.8	16.4 ± 1.9	16.3 ± 2.4	0.832
Ind. Bilirubin (mg/dL)	1.9 ± 0.5	2.0 ± 0.7	2.0 ± 0.6	0.575
White cells (x10^3^/mm^3^)	14755 ± 3095.8	14786 ± 6627.7	14543 ± 4332.8	0.975
Red cells (/mm^3^)	4.8 ± 0.6	4.4 ± 0. 5	4.6 ± 0.5	0.082
Glucose levels (mg/dL)	63.4 ± 16.3	65.8 ± 25.5	66.0 ± 20.5	0.091
Hospitalization (days)	3.0 ± 1.5	3.0 ± 1.4	**3.9 ± 3.9** ^**a,b**^	**0.005**

± *N* = 204 [54 infants with incomplete records]

* Chi-square test

# ANOVA; Tukey test

^a^ statistically different from the control group (*p* < 0.05)

^b^ statistically different from the overweight group (*p* < 0.05)

Compared to normal weight women, obese and overweight mothers had lower weight gain (WG), with odds ratio (OR) of 0.2 (0.1–0.4) and 0.4 (0.2–0.7), respectively. This indicates that overweight and obesity were protective against excess maternal weight gain. Nevertheless, obese women showed higher risk of developing hypertension (OR = 7.0; 3.1–15.9), hyperglycemic disturbances (OR = 5.5; 2.9–10.6) and HbA1c > 6.5 % (OR = 3.7; 1.2–11.1) during pregnancy. Maternal weight status did not affect neonatal outcomes (Table 5).

**Table 5:** Odds ratio (OR) and confidence interval (CI 95 %) of prepregnancy BMI *vs* maternal and neonatal outcomes

	Obese *vs* normal weight		Overweight *vs* normal weight	
	OR	CI 95 %	OR	CI 95 %
Mother				
Weight gain ≥ 16 Kg	**0.2**	**0.1 – 0.4**	**0.4**	**0.2 − 0.7**
Hypertension	**7.0**	**3.1 − 15.9**	**2.1**	**1.1 − 3.9**
Hyperglycemia	**5.5**	**2.9 – 10.6**	1.3	0.7 – 2.6
HbA1c ≥ 6.5 %	**3.7**	**1.2 – 11.1**	1.0	0.5 – 2.2
Newborn				
Weight	2.3	0.8 – 6.6	1.9	0.7 – 5.4
Weight classes	1.0	0.4 – 2.1	0.8	0.4 – 1.7
1-min Apgar score	0.4	0.1 – 1.1	0.6	0.2 – 1.5
5-min Apgar score	2.0	0.1 – 32.0	1.9	0.1 – 31.5

## Discussion

In the present study, overweight and obesity were associated with increased maternal weight, and gestational BMI, as well as higher prevalence of GDM and MGH. In the obese group, the proportion of LGA babies with disproportionate ponderal index (≥2.98), placenta weight and placental index (PI) were higher, and hospitalstay length was longer. Obesity was associated with higher risk of developing hypertension, hyperglycemia and HbA1c ≥ 6.5 % in late pregnancy. Unexpectedly, obesity and overweight were protective factors against maternal weight gain, without affecting neonatal outcomes.

Obese and overweight women were heavier and showed higher prepregnancy BMI, which was proportional to their nutritional status. These features persisted throughout pregnancy and were associated with hypertension, GDM and MGH. Similar results have also been observed in previous studies showing association of increased BMI with development of hypertension and hyperglycemia [3, 7, 22–24].

In this study, logistic regression analysis indicated obesity as a risk factor for gestational hypertension and hyperglycemia, increasing the likelihood of their occurrence seven and five-fold, respectively. This maternal characteristic was also a determining factor for the four-fold increase in the occurrence of HbA1c ≥ 6.5 % at the end of pregnancy. Hypertension and hyperglycemia associated with central obesity (in women, BMI ≥ 30 Kg/m^2^ or waist circumference ≥ 80 cm) are clinical criteria for the diagnosis of metabolic syndrome (MS), whose physiopathological basis is the association between obesity and insulin resistance [25].

Insulin resistance, classically considered as a characteristic of healthy pregnancy, is more pronounced in pregnancies complicated by diabetes [26]. The relationship between maternal MS and hyperglycemia during pregnancy was first reported by Bo et al. [27] and has been recently corroborated by our research group [28, 29]. Thus, in addition to the 13–15 % rates of pregestational DM observed in the present study, the overweight and obese groups were expected to show elevated rates of MGH or GDM, and a high proportion of HbA1c ≥ 6.5 %. However, although elevated rates of MGH (about 22 %) and GDM (approximately 30 to 40 %) were indeed observed, only 20.0 % of the overweight and obese participants showed HbA1c ≥ 6.5 %.

All overweight and obese women participating in this study received individualized dietary advice and were encouraged to engage in physical activity regardless of diagnosis of diabetes or hyperglycemia. Indeed, 51.5 and 43.3 % of overweight and obese pregnant women, respectively, adhered to physical activity recommendations, but these measures did not prevent the occurrence of MGH or GDM, probably because the period between admission and diagnostic testing for MGH/GDM was short (about four weeks). Nonetheless, there was a 60–70 % reduction in the risk of excessive weight gain, which may have contributed to the lower weight gain observed in the obese group, as well as to the significant rates of overweight and obese women (35 to 40 %) moving to lower BMI classes in late pregnancy, and the achievement of maternal hyperglycemia control (HbA1c levels < 6.5 %) in 80 % of overweight and obese women.

The relationship of diet/exercise with maternal weight remains controversial. Several studies have evaluated the effects of dietary advice alone or associated with exercise, on maternal weight gain, but no consistent results have been obtained [30–33]. A recent meta-analysis has suggested that compared to unsupervised physical activity and diet interventions, supervised exercise plus diet programs were most effective in managing weight among overweight or obese pregnant and postpartum women [34].

In this study, LGA infants, with PI ≥ 2.98 and higher thoracic and abdominal circumference measures, as well as increased placental weight and placental índex were more frequent in the obese group. According to previous studies, gestational BMI and DM or hyperglycemia are independent risk factors for excessive fetal growth [7, 12, 35, 36]. Moreover, macrosomia, disproportionate growth, placentomegaly, high placental index, and increased fetal measures (especially abdominal circumference) have been considered common features in babies born to diabetic mothers [19, 20, 35]. In Brazil, a recent review has shown that a large proportion of women are overweight or obese at the beginning of pregnancy, and that this is associated with higher risk of excessive weight gain, cesarean delivery, and fetal macrosomia [37].

In our study, overweight and obesity were identified as preventive factors against excessive weight gain without risk for the baby. Thus, the adverse neonatal outcomes here observed were probably caused by maternal hyperglycemia. Previous results obtained by our research group show that fetal macrosomia is significantly associated with prepregnancy BMI, hyperglycemic levels, and personal history of macrosomia in pregnancies complicated by diabetes or mild hyperglycemia [38]. A cohort study of 6,125 deliveries, using logistic multivariate analysis to exclude potential confounding variables, has demonstrated that only neonatal macrosomia and meconium aspiration syndrome remain significantly associated with maternal overweight and obesity [39]. Macrosomia and excessive fetal growth are frequently associated with BMI ≥ 25 kg/m^2^ and maternal diabetes or hyperglycemia, which are also interdependent. Therefore, it is practically impossible to determine the intensity of such effects.

Although indirectly, our results corroborate the usefulness of dietary advice plus exercise as a preventive intervention against excessive weight gain in pregnancies complicated by overweight or obesity. However, this study has some limitations that include the adoption of a cross-sectional rather than a randomized controlled design, the potential bias of confounding factors relative to missing personal information (pre-gestational weight, compliance to treatment protocol); and sample size, which might not have been large enough for the evaluation of neonatal outcomes and a more elaborate statistical analysis (adjustment for age, parity and maternal weight or multiple regression analysis).

## Conclusions

In the present study, adverse pregnancy outcomes were associated with obesity and overweight, and indirectly related to maternal hyperglycemia. Obesity was a determinant risk factor for hypertension, hyperglycemia, and increased HbA1c levels at the end of pregnancy. Overweight elevated the risk of hypertension, and both obesity and overweight were protective factors against excessive maternal weight gain without affecting neonatal outcomes. These findings indicate that adverse outcomes resulted from inadequate nutritional status during pregnancy, and indirectly underscore the importance of identifying and treating inadequate weight status during pregnancy.
